# Treatment of Symptomatic Non-Parasitic Liver Cysts–Surgical Treatment Versus Alcohol Injection Therapy

**DOI:** 10.1155/1990/60495

**Published:** 1990

**Authors:** Toshiya Furuta, Yasuhiro Yoshida, Motonori Saku, Hiroshi Honda, Toru Muranaka, Yoshihiko Oshiumi, Takashi Kanematsu, Keizo Sugimachi

**Affiliations:** 1 Departments of Surgery and Radiology National Fukuoka Central Hospital Fukuoka Japan; 2 Department of Surgery II Faculty of Medicine Kyushu University Fukuoka Japan; 3 Department of Surgery II Faculty of Medicine Kyushu University Fukuoka 812 Japan

## Abstract

Fourteen patients with benign symptomatic non-parasitic cysts of the liver were either surgically treated, had alcohol injected into the cysts, underwent deroofing of the cyst or in 5, a cystectomy was done. Alcohol was injected into 6 patients and there has been no recurrence for as long as 5 years and 8 months after the treatment. Liver dysfunction occurred in 3 patients given blood transfusion during the surgery and/or postoperative course, an elevated temperature (over 39℃) occurred in one patient. Adverse effects of alcohol injections were minor and transient. Based on our experience, the injection of alcohol is an effective treatment for benign symptomatic cyst of the liver. When a malignancy is suspected on imaging and/or cytologic studies, or when alcohol administration is ineffective, then surgery is indicated.


*HPB Surgery*, 1990, Vol. 2, pp. 269–279
Reprints available directly from the publisher
Photocopying permitted by license only

© 1990 Harwood Academic Publishers GmbH
Printed in the United Kingdom

### TREATMENT OF SYMPTOMATIC NON-PARASITIC LIVER CYSTS — SURGICAL TREATMENT VERSUS ALCOHOL INJECTION THERAPY

TOSHIYA FURUTA*, YASUHIRO YOSHIDA, MOTONORI SAKU, HIROSHI HONDA, TORU MURANAKA, YOSHIHIKO OSHIUMI, TAKASHI KANEMATSU and KEIZO SUGIMACHI

*Departments of Surgery and Radiology, National Fukuoka Central Hospital, Fukuoka, and Department of Surgery II, Faculty of Medicine, Kyushu University, Fukuoka, Japan*

Fourteen patients with benign symptomatic non-parasitic cysts of the liver were either surgically treated, had alcohol injected into the cysts, underwent deroofing of the cyst or in 5, a cystectomy was done. Alcohol was injected into 6 patients and there has been no recurrence for as long as 5 years and 8 months after the treatment. Liver dysfunction occurred in 3 patients given blood transfusion during the surgery and/or postoperative course, an elevated temperature (over 39°C) occurred in one patient. Adverse effects of alcohol injections were minor and transient. Based on our experience, the injection of alcohol is an effective treatment for benign symptomatic cyst of the liver. When a malignancy is suspected on imaging and/or cytologic studies, or when alcohol administration is ineffective, then surgery is indicated.

KEY WORDS: Non-parasitic liver cyst, deroofing, alcohol injection therapy

### INTRODUCTION

Nonparasitic liver cysts, once considered to be a rare occurrence are now being detected incidentally during ultrasonography (US) and computed tomography (CT). Although commonly asymptomatic, these cysts may be accompanied by symptoms of discomfort, abdominal mass, hepatomegaly or abdominal pain[1] and there is a risk of rupture[2] if the cyst becomes enlarged. Resection,[3] deroofing[4] and fenestration[5] can be done to treat these non-parasitic liver cysts. Bean and co-workers reported that the direct injection of alcohol into renal[6] (1981) and liver cysts[7] (1985) was an effective treatment. Between 1980 and 1988, symptomatic non-parasitic liver cysts were treated by deroofing and cystectomy, and alcohol injection therapy for liver cysts was introduced in 1984 at the National Fukuoka Central Hospital and at Kyushu University. We report here the long term results of this treatment and the indication for these modes of therapy is discussed.

### MATERIALS AND METHODS

Between May 1980 and April 1988, fourteen patients with symptoms of benign non-parasitic liver cysts were admitted to National Fukuoka Central Hospital or to

*Correspondence and reprint requests to: Toshiya Furuta MD, Department of Surgery II, Faculty of Medicine, Kyushu University, Fukuoka 812, Japan.

Kyushu University Hospital (Table 1). All 14 were women, aged 40–75 (average 60). Symptoms were abdominal discomfort in 7, upper abdominal pain in 6 and an abdominal mass in 1. Solitary liver cysts were identified in 8 and one patient had a coexisting renal cyst. Multiple liver cysts were present in the other 6 patients and one had a coexisting pancreatic cyst. Diagnosis of liver cyst was made using US and CT. Drip infusion cholangiography was performed before CT to demonstrate any communication between the liver cyst and the biliary tree (DIC-CT). Skin and antibody tests were performed to exclude parasitic causes of the liver cysts. Cytologic and bacteriologic evaluations were made of the cyst fluid.

Treatment of liver cysts included surgery, and alcohol injection into the cyst which was introduced in 1984 in our institutes. Since then, alcohol injection has been our treatment of choice. Surgical intervention is considered when (1) alcohol injection is ineffective, (2) alcohol injection is technically difficult, (3) malignancy is suspected, (4) there is a communication between the cyst and the biliary trees. The alcohol was injected by internists following discussion with our surgical teams. In 3 patients in the present study who underwent deroofing, the dome of the cyst was excised and the yellow fluid of the cyst was drained, leaving its base attached to the liver. In 5 treated by cystectomy, resection of the cystic portions of the liver was done and the normal tissue was left untouched. Histological examination of the resected specimens was done.

In 6 patients, alcohol was instilled into the liver cyst. An 18 gauge needle was passed into the cyst, under the guidance of US. The initial aspiration of fluid was observed for color and viscosity, then cytologic and bacteriologic evaluations were made. A J shaped-guide wire was placed through the needle and into the cyst, and the needle was withdrawn. A #5-French pigtail catheter was placed in the cyst over the guide-wire. After complete aspiration of the fluid, contrast material was injected into the cyst cavity to ensure that there was no communication with the biliary tree or vessels. After complete aspiration of the contrast medium, 100–150 ml/body of sterile absolute alcohol was instilled through the catheter and the catheter was left in place for 5–20 minutes, during which time the patient was rolled about into various positions so that the alcohol would make contact with all of the cystic wall. As only one instillation of alcohol did not obliterate the cyst in some patients, the catheter was left in situ for additional injections of alcohol. If excretion from the catheter continued, about 50 ml/body of additional alcohol was given about seven days after the first instillation, as required. The catheter was removed when all secretion ceased.

Follow-up with US and CT was done for all 14 patients.

### RESULTS

Three patients who underwent the deroofing procedure in 1983 complained of abdominal discomfort preoperatively and there were no remarkable changes in the laboratory data. Two patients had a solitary liver cyst (8. 16 cm) in the right and left hepatic lobe, the other one had multiple liver cysts in both hepatic lobes. CT, after admission, of the first case revealed a 8 cm cyst in the right hepatic lobe (Figure 1a). Cytologic examination of the cyst fluid showed all these cysts to be benign. In all three cases, the liver cysts disappeared after treatment and there were no postoperative complications. There has been no recurrence on the follow-up CT examinations in these three for as long as 6 years and 9 months. CT taken four years after surgery for the case 1 patient showed marked improvement (Figure 1b).

**Table 1** Results of various treatments for non-parasitic liver cysts.

| | | | *Liver cyst* | | | | | | |
|---|---|---|---|---|---|---|---|---|---|
| *Case* | *Age/Sex* | *Symptom* | *Location* | *Type* | *Size (cm)* | *Volume (ml)* | *Treatment* | *Complication* | *Periods after treatment*[b] |
| 1 | 51/F | discomfort | right | S | 8 | 200 | Deroofing | none | 6Y 9M |
| 2 | 73/F | discomfort | bilateral | M | 9 | 1150 | Deroofing | none | 6Y 5M |
| 3 | 68/F | discomfort | left | S | 16 | 500 | Deroofing | none | 6Y 3M |
| 4 | 61/F | pain | right | M | 9 | 500 | Cystectomy | liver dysfunction | 9Y 5M |
| 5 | 40/F | pain | left | S | 12 | 500 | Cystectomy | liver dysfunction | 7Y 2M |
| 6 | 71/F | mass | bilateral | M | 22 | 650 | Cystectomy | liver dysfunction dysfunction | 5Y 3M |
| 7 | 56/F | discomfort | bilateral | M | 10 | 2000 | Cystectomy | fever | 3Y 6M |
| 8[a] | 53/F | discomfort | right | S | 14 | 1065 | Alcohol Cystectomy | none | 1Y 2M |
| 9 | 75/F | pain | left | S | 9 | 320 | Alcohol | chest pain | 5Y 8M |
| 10 | 51/F | pain | left | S | 12 | 260 | Alcohol | fever flushing | 5Y 1M |
| 11 | 57/F | discomfort | right | S | 15 | 1520 | Alcohol | chill | 4Y 7M |
| 12 | 61/F | pain | bilateral | M | 5 | 215 | Alcohol | inebriation | 5Y 7M |
| 13 | 69/F | pain | right | S | 17 | 440 | Alcohol | inebriation | 5Y |
| 14 | 50/F | discomfort | bilateral | M | 7 | 120 | Alcohol | flushing | 3Y 6M |

S: Solitary. M: Multiple.

[a] Alcohol injection followed by cystectomy.

[b] All patients are living with no evidence of recurrence at this writing.

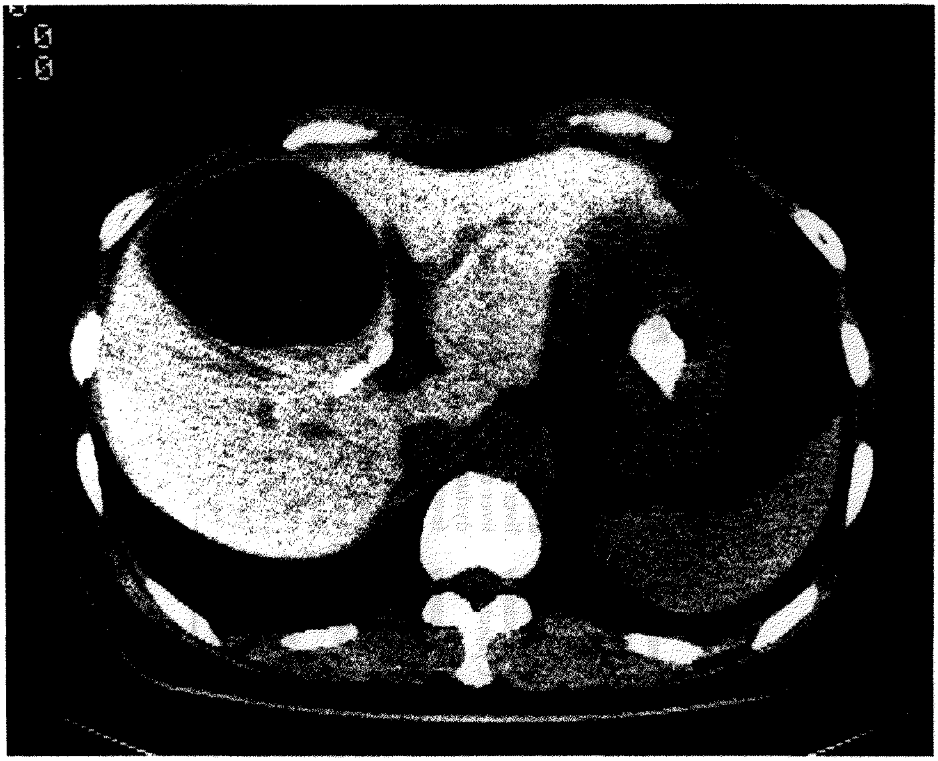

**Figure 1a** Preoperative CT showed a 8 cm cyst in the right hepatic lobe (Case 1).

In five patients treated with cystectomy, the indocyanine green 15-minute retention test was 21.3% in the case 4 patient and in the other 4 patients, there were no remarkable changes in the laboratory data. Two patients had a solitary liver cyst (12, 14 cm) in the right hepatic lobe, 2 had multiple liver cysts in bilateral hepatic lobes and one had multiple liver cysts in the right hepatic lobe. In the case 8 patient, six months before surgery, 1065 ml cyst fluid was drained and 100 ml alcohol was instilled into the cyst. The cyst decreased from 14 to 10 cm in diameter six days after treatment but again increased to 12 cm 27 days after the treatment. As the injection of alcohol was ineffective in treating this patient, surgery was performed. The cysts ranged in volume from 500 to 2000 ml in size from 9 to 22 cm. Histological and cytological examinations of the five liver cysts showed benign lesions. In all five patients, the liver cysts disappeared after treatment. Postoperative complications were liver dysfunction in 3 and high fever (over 39°C) in one patient. There has been no recurrence for as long as 9 years and 5 months.

In six patients treated by sonographically guided aspiration and alcohol injection, four patients had a solitary liver cyst and the other two multiple liver cysts. The diameter of the cysts ranged from 5 to 17 cm and the volume was from 120 to 1520 ml. CT in the case 11 patient revealed a 15 cm cyst involving the entire right hepatic

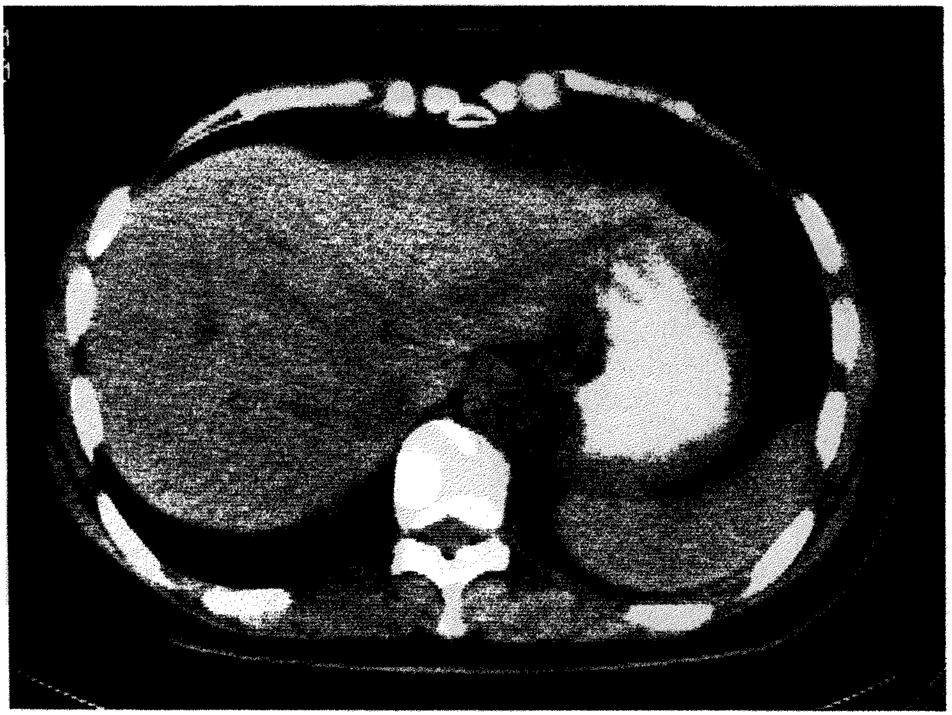

**Figure 1b** CT four years after deroofing showed the marked improvement (Case 1).

lobe (Figure 2a). All fluid cytologies and cultures were negative and the liver function tests were within normal limits. Cystography showed no communication between the liver cyst and biliary trees or vessels (Figure 2b). 100–150 ml alcohol was instilled into the liver cyst. As excretion from the catheter continued, additional injections of alcohol were performed, twice for three patients at seven days after the first treatment (cases 10, 11, 14) and five times for one patient at 1, 3, 7 and 10 days after the first treatment (case 13). In 3 of 6 patients, the liver cysts disappeared and in the other three, size of the cysts decreased and the symptoms subsided. In all 6, minor complications of chest pain, flushing, fever (37.4°C), chills and a feeling of inebriation occurred, but all symptoms disappeared two or three hours later. There were no major complications. CT taken four years after the treatment in the case 11 patient revealed a marked decrease in the liver cyst with little remaining fluid (Figure 2c). Recurrence on a follow-up CT and US in 6 patients has been nil for as long as 5 years and 8 months.

### DISCUSSION

The classification of liver cysts as proposed by Henson *et al.*[8] is based essentially on

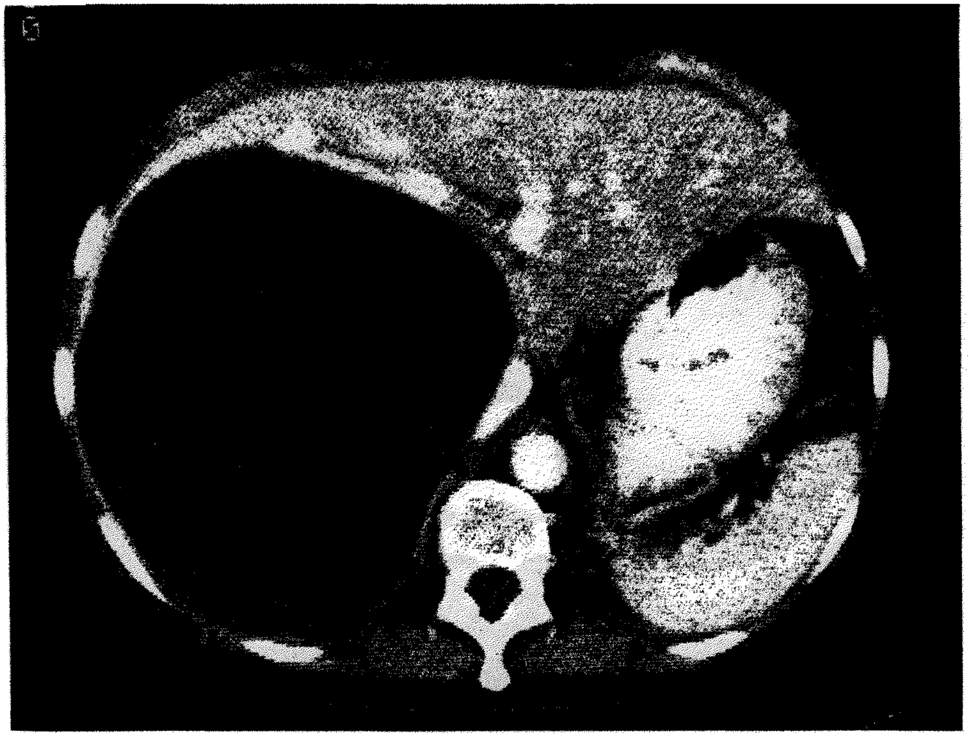

**Figure 2a** CT before aspiration revealed a 15 cm cyst in the right hepatic lobe (Case 11).

congenital, traumatic, inflammatory and neoplastic origins. With the extensive application of US and CT, asymptomatic congenital hepatic cysts are being increasingly identified[9]. The great majority of patients have congenital hepatic cysts which are small and asymptomatic, but occasionally large cysts may form a mass in the upper abdomen and vague gastrointestinal discomfort ensues. Asymptomatic liver cysts do not require treatment, there is no evidence of a malignancy and spontaneous cyst rupture does not occur. The occurrence of a carcinoma in solitary non-parasitic cysts of the liver is rare,[10] as is the complication of spontaneous cyst rupture[11]. In our patients, the hepatic cysts measured 5 to 22 cm and all patients had some symptoms. Only those with benign, non-parasitic liver cysts were treated, regardless of the size of the cyst. Haded *et al.*[2] suggested that laparotomy was indicated for patients with symptoms or an uncertain diagnosis and definitive surgical treatment was indicated only for cysts larger than 10 cm.

Various authors have reported adequate methods of treating symptomatic nonparasitic liver cysts, including: simple aspiration,[12] marspialization, external drainage, internal drainage into a jejunal loop,[13] fenestration, deroofing and complete excision of the cyst. In our patients, a communication between the cyst and the biliary system was not confirmed, using DIC-CT. If the cyst is benign and the content of the cyst is clear, the deroofing procedure is effective and a recurrence

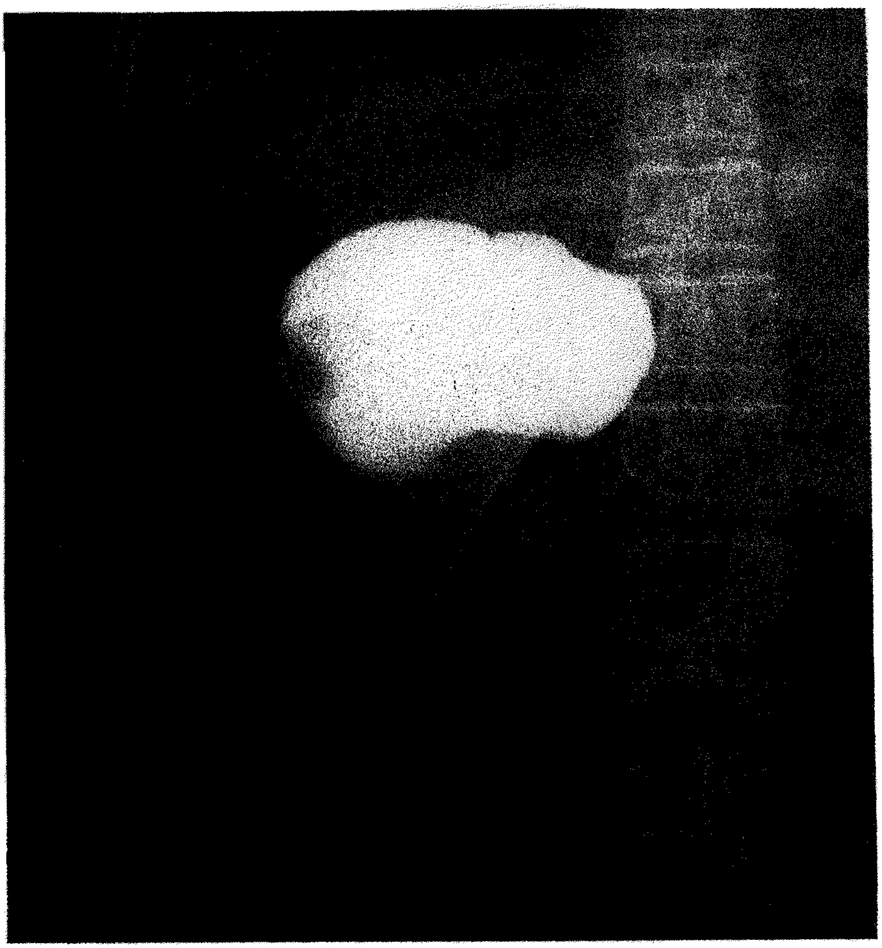

**Figure 2b** After percutaneous aspiration, contrast material was instilled into the cyst cavity. Extravasation or biliary communications were absent (Case 11).

is rare. This procedure is simple and the operation time is short. A preoperative diagnosis for malignancy of a hepatic cyst is difficult to make, but if a malignancy is suspected by cytology of the aspirated cyst fluid, then angiography might be required to examine the neovascularity.

The first description of instillation of sclerosing agents into the hepatic cyst was made by Goldstein *et al.*[14] in 1976.. While they used pantopaque, other surgeons injected alcohol into renal and orbital cysts[15]. Bean and Rodan[7] reported that six patients with hepatic cysts were successfully treated with percutaneous aspiration and injection of alcohol into the cyst. Absolute alcohol acts as a thromboembolic agent when given by direct intravascular injection[16]. The alcohol fixes the cells and they no longer secrete fluid. The optimal dose and period of the injection of the

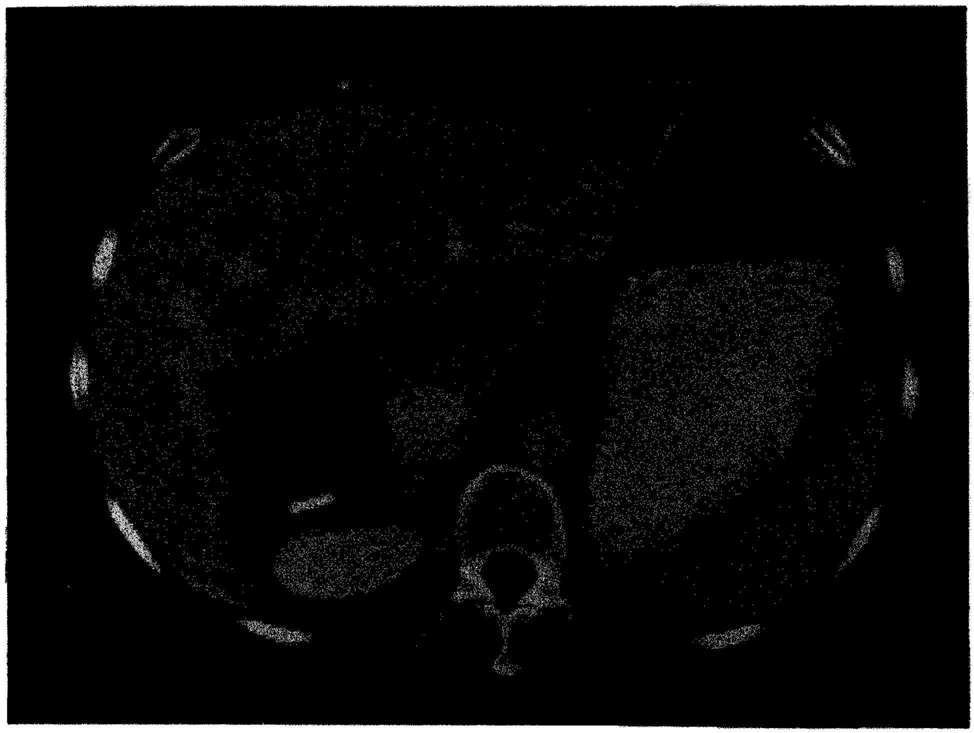

**Figure 2c** CT four years after treatment with alcohol revealed a marked decrease in size of the cyst (Case 11).

alcohol for hepatic cyst is not clearly defined. For our patients, we gave 100–150 ml/body of absolute alcohol for over a 5–20 minute period. Cystography was performed to confirm the communication between the cyst cavity and vessels or biliary systems. There were no major complications and no liver dysfunction after this instillation of alcohol. As only one instillation of alcohol may not lead to a uniform contact all of the cystic wall cells, additional injections may have to be given for a cure. In our six patients, follow-up US and CT revealed no evidence of recurrent cysts for as long as 5 years and 8 months, at this writing.

Alcohol injection therapy is technically simple and effective and is without major complications and recurrence. Post-treatment complications of alcohol injection therapy were few. The diagnosis of a benign liver cyst should be based on evidence obtained using US, CT and angiography or cytology of the cyst fluid. If a malignancy is suspected and if alcohol injection therapy is ineffective, then a complete resection of the cyst should be done. In cases where a malignancy is suspected, direct biopsy of the cyst wall is strongly recommended for an appropriate surgical design.

*Acknowledgment*

We thank M. Ohara for helpful comments.

### References

1. Sanfelippo, P.M., Beahrs, O.H. and Weiland, L.H. (1974) Cystic disease of the liver. *Annals of Surgery*, **179**, 922–925
2. Hadad, A.R., Westbrook, K.C., Graham, G.G., Morris, W.D. and Campbell, G.S. (1977) Symptomatic nonparasitic liver cysts. *American Journal of Surgery*, **134**, 739–744
3. Armitage, N.C. and Blumgart, L.H. (1984) Partial resection and fenestration in the treatment of polycystic liver disease. *British Journal of Surgery*, **71**, 242–244
4. Belcher, H.V. and Hull, H.C. (1969) Nonparasitic cysts of the liver: Report of three cases. *Surgery*, **65**, 427–431
5. Lin, T.Y., Chen, C.C. and Wang, S.M. (1968) Treatment of non-parasitic cystic disease of the liver: a new approach to therapy with polycystic liver. *Annals of Surgery*, **168**, 921–927
6. Bean, W.J. (1981) Renal cysts: treatment with alcohol. *Radiology*, **138**, 329–331
7. Bean, W.J. and Rodan, B.A. (1985) Hepatic cysts: treatment with alcohol. *American Journal of Roentgenology*, **144**, 237–241
8. Henson, S.W., Gray, H.K. and Dockerty, M.B. (1957) Benign tumors of the liver, IV. Polycystic disease of surgical significance. *Surgery Gynecology and Obstetrics*, **104**, 63–67
9. Solgaard, S., Burcharth, F., Miskowiak, J., Grønvall, S. and Hancke, S., (1984) Ultrasonically diagnosed simple cysts of the liver. *Radiologe*, **24**, 195–197
10. Bloustein, P.A. (1977) Association of carcinoma with congenital cystic conditions of the liver and bile ducts. *American Journal of Gastroenterology*, **67**, 40–46
11. Morgenstern, L. (1959) Rupture of solitary nonparasitic cysts of the liver. *Annals of Surgery*, **150**, 167–171
12. Flagg, R.S. and Robinson, D.W. (1967) Solitary nonparasitic hepatic cysts: report of oldest known case and review of the literature. *Archives of Surgery*, **95**, 964–973
13. Longmire, W.P., Mandiola, S.A. and Gordon, H.E. (1971) Congenital cystic disease of the liver and biliary system. *Annals of Surgery*, **174**, 711–726
14. Goldstein, H.M., Carlyle, D.R. and Nelson, R.S. (1976) Treatment of symptomatic hepatic cyst by percutaneous instillation of pantopaque. *American Journal of Roentgenology*, **127**, 850–853
15. Hornblass, A. and Bosniak, S. (1981) Orbital cysts following enucleation: the use of absolute alcohol. *Ophthalmic Surgery*, **12**, 123–126
16. Ellman, B.A., Green, C.E., Eigenbrodt, E., Garriott, J.C. and Curry, T.S. (1980) Renal infarction with absolute ethanol. *Investigative Radiology*, **15**, 318–322

(Accepted by S. Bengmark on 22 October 1989)

### INVITED COMMENTARY

The manuscript by Dr. Furuta and colleagues on "treatment of symptomatic non-parasitic liver cysts — surgical treatment versus alcohol injection therapy" is an excellent contribution in the field of treatment of liver cysts. We have a similar experience in the treatment of single non-parasitic cysts with alcohol therapy recently published in the British Journal of Surgery. A few points stressed in the articles are worthy of comment. First, it is important to use a pig tail catheter placed in the cyst cavity to avoid leakage of alcohol into the abdominal cavity which may occur and cause pain for the patient. Second, the authors are careful to document that there is no communication to the biliary tree which is very important if damage to the biliary system is to be avoided. Third, it is essential to know that these cysts are not parasitic because then the treatment is different. I think this therapy is now generally approved in the treatment of these types of cysts. The problem is how to treat patients with multiple cysts. In my opinion these are the patients which are mostly symptomatic and they create therapeutic problems. We have also in this group of patients used alcohol sclerotherapy but we do not know how much alcohol to use on single injections and also if there are other potential side-effects on long-term follow-up after repeated injections. Maybe Dr Furuta and colleagues will come back to this problem in the future. They ought to be congratulated on a nice paper.

B. Jeppsson
Department of Surgery
Lund University
Lund

### INVITED COMMENTARY

We are pleased to have been given the opportunity to comment on Dr. Furuta's publication supporting the use of alcohol injection therapy for symptomatic non-parasitic liver cysts. The results of his alcohol injection therapy appear to be very promising, in that an apparently effective treatment is followed by amelioration of symptoms, and few complications. Our own experience with wide unroofing[1] led us to believe that "conservative" surgical management was better than previously reported Roux-Y drainage or cystectomy; Dr. Furuta's method may well be an even more conservative and acceptable treatment.

Although the alcohol injection series is a consecutive one with followup that is necessarily shorter than the surgical series, the followup seems adequate to suggest a positive long-term effect. However it may be a mistake to compare it to surgically treated cases in a non-randomized fashion.

We are always concerned about the possibility of malignancy in hepatic cysts. One of our 22 cases had a cystadenocarcinoma, and biopsy of the cyst wall at surgery was therefore critical. This diagnosis would have been difficult to make on cytology only. It is well known that atypical features such as septations, solid elements, irregularity, thickening, or calcification of the cyst wall should be noted preoperatively on imaging, suggesting a disease process other than simple cysts.[2,3]

Of the six patients receiving alcohol injection therapy in Dr. Furuta's series, three patients received two injections, and one patient received five injections with a catheter being left continuously in the cyst for the course of the injection treatments. Although there was no incidence of significant sepsis in these cases, we would be concerned that routine use of such long-term indwelling catheters (up to 10 days) would inevitably lead to a significant incidence of sepsis.

Finally, Dr. Furuta implies that the treatment failure should be treated by cystectomy including removal of all lining. Whether resection of the whole cyst lining includes formal liver resection or not, a significant incidence of complications has been reported[4,5]; we have found that wide unroofing of the easily accessible cyst without the necessity of removing all cyst lining attached to the liver, is adequate treatment.[1]

Alcohol injection therapy of simple hepatic cysts shows real promise, and deserves further comparison to surgical alternatives such as wide unroofing.

Bryce Taylor
Toronto General Hospital

### *References*

1. Litwin D.E.M., Taylor B.R., Greig P., Langer B. (1987) Non-parasitic cysts of the liver — the case for conservative surgical management. *Annals of Surgery*, Vol 205(1):45–48.
2. Hattner R.S., Engelstad B.L. (1983) Diagnostic imaging and quantitative physiologic function using radionuclide techniques in gastrointestinal disease. In Sleisinger M.H., Fordtran J.S. (editors) Gastrointenstinal Disease, p. 1667. Toronto: W.B. Saunders.
3. Roemer C.E., Ferrucci J.T., Mueller P.R., *et al.* (1981) Hepatic cysts: diagnosis and therapy by sonographic needle aspiration. *AJR*, **136**, 1065.
4. Coutosoftides T., Hermann R.E. (1974) Non-parasitic cysts of the liver. *Surgery Gynecology and Obstetrics*, **138**, 906–13.
5. Longmire W.P., Mandiola S.A., Gordon H.E. (1971) Congenital cystic disease of the liver and biliary system. *Annals of Surgery*, **174**, 711.

